# A Case Report of New-Onset Diabetes Mellitus as an Early Warning Sign of Pancreatic Ductal Adenocarcinoma in an Elderly Patient: The Earlier the Diagnosis and Surgery, the Better the Prognosis

**DOI:** 10.7759/cureus.31608

**Published:** 2022-11-17

**Authors:** Michele Bertoni, Costanza Bertoni, Silvia Abatangelo, Marco Scatizzi, Pamela Lotti

**Affiliations:** 1 2nd Unit of Internal Medicine, Ospedale Santo Stefano, Prato, ITA; 2 Unit of Infectious Diseases, Università Vita-Salute San Raffaele, Milano, ITA; 3 Unit of General Surgery, Ospedale Santa Maria Annunziata, Firenze, ITA; 4 Unit of High Intensity Internal Medicine, Ospedale Santo Stefano, Prato, ITA

**Keywords:** pancreatoduodenectomy, pancreatic ductal adenocarcinoma, ca 19.9, pancreatic cysts, new-onset diabetes mellitus

## Abstract

Studies have been recently conducted to find pancreatic ductal adenocarcinoma (PDAC) in high-risk groups by identifying individuals with pancreatic cystic lesions and elderly people (> 50 years) with new-onset diabetes mellitus (NODM). We report the case of a 91-year-old woman in good health with pancreatic cysts, who firstly displayed a NODM and, one month later, a PDAC. A dehydration syndrome with polydipsia and asthenia led to her hospitalization. High levels of blood glucose and glycated hemoglobin were found. An abdomen US showed a minute pancreas with some cysts. Rehydration and insulin therapy led to a good glycemic compensation. One month after discharge, she displayed weight loss, diarrhea, and jaundice. On the second admission, high levels of total and direct bilirubin, indices of hepatic cholestasis, and Ca 19.9 were found. An abdomen contrast medium CT evidenced a nodule at the pancreatic head, suggesting a malignant lesion, and dilatation of both the Wirsung duct and the whole biliary tract. Despite the lack of a histological diagnosis, the absence of signs of local invasion, metastasis, and co-morbidities as well as the rapid clinical deterioration led us to propose surgical treatment. A few days later, a pancreatoduodenectomy was performed. The histologic examination showed a moderately differentiated (G2) PDAC. The TNM staging was IIA (pT3, N0, M0) (R0). Three weeks after, she was discharged with normal liver function tests, Ca 19.9 levels, and a good glycemic compensation with insulin therapy. Five years after surgical treatment, she is still doing well displaying a normal abdomen CT follow-up, normal blood tests, including Ca 19.9, and a good glycemic compensation. Our case report denotes an exceptional duration of survival of PDAC and highlights the importance to seek its presence in every case of NODM in patients > 50 years, especially if they carry pancreatic cysts.

## Introduction

Pancreatic ductal adenocarcinoma (PDAC) is most frequently detected at an advanced stage. Such late detection restricts treatment options and contributes to a poor five-year survival rate of 3-15% [1,2]. Studies have been recently conducted to find PDAC in high-risk groups by identifying individuals in families with an inherited risk [3,4], people with cystic lesions of the pancreas [5,6], and elderly people ( > 50 years) with new-onset diabetes mellitus (NODM) [7,8]. Concerning pancreatic cysts, they are found in approximately 8% of individuals older than 70 years [9]. In such individuals, the intraductal papillary mucinous neoplasms (IPMN) and the mucinous cystic neoplasms can be precursors to PDAC in up to 15% of the cases. Such cysts are collectively referred to as mucinous cystic lesions and are incidentally found in 3% of individuals undergoing a CT scan [10]. With regard to NODM, compared with the general population, in individuals with type 2 diabetes mellitus (DM) for less than one year, the relative risk of PDAC increases four to five-fold with respect to people with long-standing type 2 DM (more than five years) who have a 1-1.5-fold increased relative risk [11]. Here, we report the case of an elderly woman with pancreatic cysts who presented with NODM, which was followed by the early appearance of a PDAC.

## Case presentation

The patient was a 96-year-old woman with a healthy lifestyle and no current comorbidities, whose medical history was only characterized by an episode of paroxysmal atrial fibrillation and ischemic colitis in 2011. At the time of the first hospitalization (February 2017), she was taking only clopidogrel 75 mg once daily and had a high Karnofsky Performance Scale Index (KPSI) (90). She suddenly displayed a dehydration syndrome with polydipsia and asthenia. Her physical examination was not remarkable except for signs of mucosal and skin dehydration. Her body mass index (BMI) was 27.2 kg/m^2^. On admission, high blood glucose and glycated hemoglobin (HbA1c) levels along with 500 mg/dL glycosuria on spot urine specimen, were found. The most significant blood tests of the patient are reported in Table 1.

**Table 1:** Most significant blood tests in the first admission HbA1c: Glycated hemoglobin; LDL: Low Density Lipoprotein; ALT: Alanin Transaminase; AST: Aspartate Transaminase; Gamma-GT: Gamma-Glutamyl Transpeptidase

Test	Results	Reference values
Fasting blood glucose	397 mg/dL	65-110 mg/dL
HbA1c	92 mmol/mol	20-40 mmol/mol
C peptide	0.98 ng/mL	0.6-4.4 ng/mL
Total cholesterol	118 mg/mL	< 200 mg/mL
LDL cholesterol	75 mg/dL	< 100 mg/dL
Triglycerides	130 mg/dL	< 150 mg/dL
Uric acid	3.7 mg/dL	2.6–6.0 mg/dL
Total bilirubin	0.7 mg/dL	< 1.1 mg/dL
Direct bilirubin	0.2 mg/dL	< 0.25 mg/dL
ALT	14 UI/L	< 40 UI/L
AST	21 UI/L	< 40 UI/L
Alkaline phosphatase	127 UI/L	30-130 UI/L
Gamma-GT	53 UI/L	< 38 UI/L
Hemoglobin	13.4 g/dL	12-16 g/dL
Creatinine	0.68 mg/dL	0.44-0.95 mg/dL

**Figure 1:**
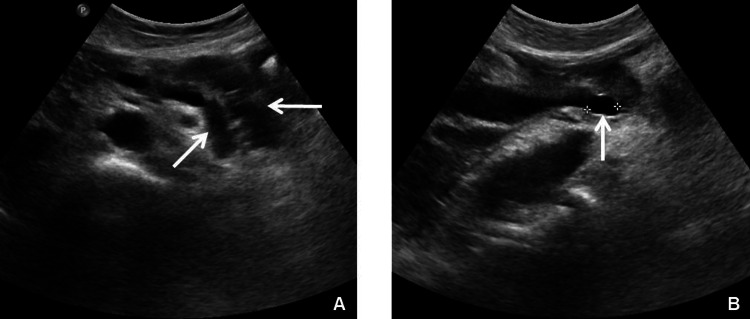
Abdominal ultrasound of February 2017 (A) Horizontal arrow indicates reduced dimensions of the pancreas. Oblique arrow indicates dilatation of Wirsung duct. (B) Vertical arrow indicates the pancreatic cyst with 13 mm diameter at its head.

An abdomen ultrasound (US) showed reduced dimensions of the pancreas with dilatation of the Wirsung duct (Figure 1A) and some cysts in the head and body, the largest of which had a 13 mm diameter (Figure 1B). In addition, the choledochus had a 12 mm diameter without stones in the lumen and the gallbladder contained a 15 mm stone with corpuscular-looking bile. 

**Figure 2:**
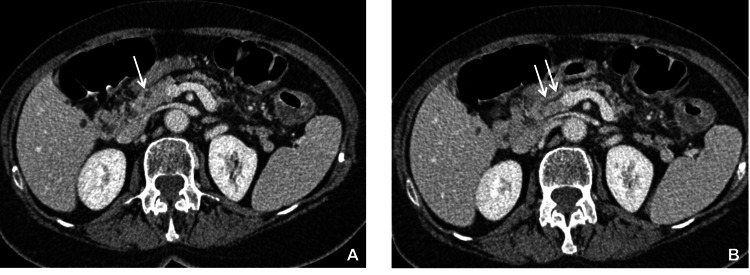
Abdominal contrast medium computed tomography of 2011 (A) Arrow indicates a minute cyst in the head of the pancreas, suspected for type1-IPMN, that is, in relation with the Wirsung duct. (B) The two arrows indicate a slightly dilated Wirsung duct IPMN: intraductal papillary mucinous neoplasm

The comparison with an abdomen contrast medium CT (CMCT) of 2011 (carried out during the hospitalization for ischemic colitis) showed that, at the time, there was a minute cystic form of 6 mm diameter in the head of the pancreas (Figure 2A), near a slightly dilated Wirsung duct (Figure 2B). Such cyst was suspected for type 1-intraductal papillary mucinous neoplasm (IPMN), that is, in relation to the Wirsung duct. 

The patient was treated with rehydration and insulin therapy, which led to a good glycemic compensation. After eight days she was discharged.

During the following three weeks, she began to experience weight loss (3 kg), diarrhea with four-five discharges/day of hypocholic stools, hyperchromic urine, and, finally, jaundice. She was immediately hospitalized and her physical examination evidenced jaundice, icteric sclerae, and the positivity of the Courvoisier-Terrier sign. Her BMI was 26 kg/m^2^. Concerning the blood tests, high levels of total and direct bilirubin, bile acids, indices of intra- and extra-hepatic cholestasis, and Ca 19.9 were found along with a marked reduction of C peptide values, suggestive of pancreatic atrophy. Conversely, the glycemic compensation with insulin therapy was good. The most significant blood tests of the second admission are reported in Table 2.

**Table 2:** Most significant blood tests in the second admission LDL: Low-Density Lipoprotein; ALT: Alanine Transaminase; AST: Aspartate Transaminase; Gamma-GT: Gamma-Glutamyl Transpeptidase; Ca 19.9: Carbohydrate antigen 19-9; HbA1c: Glycated hemoglobin

Test	Results	Reference values
Total bilirubin	8.7 mg/dL	< 1.1 mg/dL
Direct bilirubin	4.5 mg/dL	< 0.25 mg/dL
ALT	77 UI/L	< 40 UI/L
AST	100 UI/L	< 40 UI/L
Alkaline phosphatase	614 UI/L	30-130 UI/L
Gamma-GT	312 UI/L	< 38 UI/L
Bile acids	50.6 µmol/L	< 6 µmol/L
Ca 19.9	2263 UI/mL	< 38 UI/ml
Fasting blood glucose	97 mg/dL	65-110 mg/dL
HbA1c	55 mmol/mol	20-40 mmol/mol
C peptide	0.58 ng/mL	0.6-4.4 ng/mL
Total cholesterol	363 mg/mL	< 200 mg/dL
LDL cholesterol	75 mg/dL	< 100 mg/dL
Triglycerides	145 mg/dL	< 150 mg/dL
Hemoglobin	13.4 g/dL	12-16 g/dL
Creatinine	0.59 mg/dL	0.44-0.95 mg/dL

**Figure 3:**
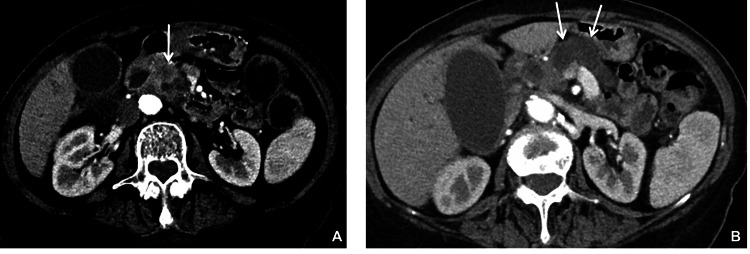
Abdominal contrast medium computed tomography of 2017 (A) Vertical arrow indicates a suspected malignant neoplastic nodule at the pancreas head. (B) The two arrows indicate Wirsung duct ectasia with almost complete atrophy of the pancreatic parenchyma.

A total body CMCT evidenced the following alterations: (i) at the pancreas head, a nodule of inhomogeneous tissue with a diameter of about 18 mm, suggestive of a malignant neoplastic lesion (Figure 3A), (ii) Wirsung duct ectasia (diameter of 18 mm) with almost complete atrophy of the residual pancreatic parenchyma (Figure 3B), (iii) ectasia of the intra- and extrahepatic biliary tract, in particular of the choledochus, showing a 20 mm diameter up to the proximity of the retropancreatic tract, and (iv) marked ectasia of the gallbladder, with some non-obstructing stones in the lumen. The liver was free from secondary focal lesions. Moreover, there were no signs of invasion of the surrounding vascular structures. Endo- and retroperitoneal lymph node swellings as well as significant changes in the lung parenchyma, pleura, and airways, were absent. Interestingly, the comparison with the abdomen CMCT of 2011 showed a correspondence between the site of the suspected neoplastic lesion and that of the previous type 1-IPMN (compare Figure 2A with Figure 3A).

On the whole, despite the lack of a histological diagnosis, the aforementioned data along with the rapid worsening of jaundice, weight loss, and total and direct bilirubin values strongly argued in favor of a diagnosis of pancreatic cancer. 

The decision on its management was taken by a multidisciplinary specialist team consisting of a gastroenterologist, a surgeon, a radiologist, an oncologist, and an internist. Taking into account the absence of signs of local invasion, metastases, and co-morbidities, the good glycemic compensation, and the high KPSI, it was proposed surgical treatment according to the recommendation 2.1 of the American Society of Clinical Oncology Clinical Practice Guideline [12]. The patient, once informed of the risks of surgery and its possible complications, gave written consent. The surgical intervention consisted of the bi-subcostal incision for access, release of the head and pancreatic hook from the retroportal lamina, radical Traverso-Longmire pylorus-preserving pancreatoduodenectomy, cholecystectomy, removal of the common bile duct and choledochectomy, hepatic-jejunal anastomosis (with placement of drainage), and termino-lateral duodenal-jejunal anastomosis. The proximal 2 cm of the duodenum was preserved.

**Figure 4:**
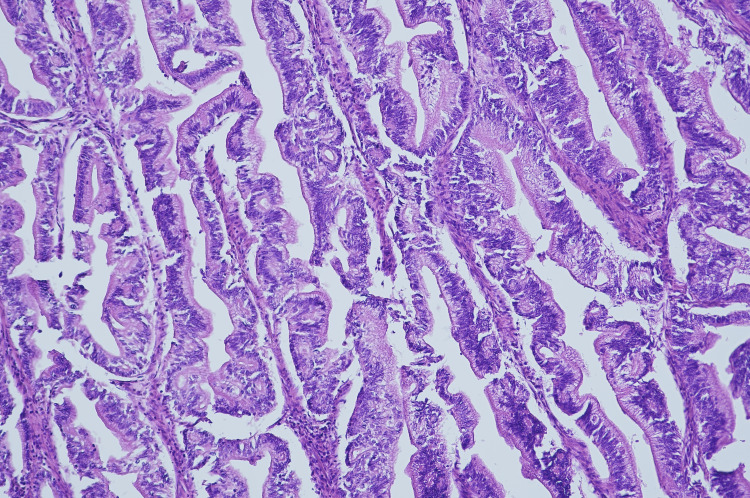
Hematoxylin-eosin stained section of the patient's moderately differentiated (G2) pancreatic ductal adenocarcinoma (40 x).

The histopathological examination of the surgical specimen macroscopically showed a solid neoplasm with a diameter of 15 mm of the head of the pancreas, infiltrating the duodenal wall and the papilla of Vater. The gallbladder was macroscopically free of pathological changes. At the microscopic level, it was evidenced a moderately differentiated (G2) PDAC (Figure 4).

The postoperative course was characterized by monitoring drainage, resuming nutrition, and mobilization. Three weeks after admission, she was discharged with markedly improved liver function tests, normal Ca 19.9, and good glycemic compensation with insulin therapy. The most significant blood tests carried out at the end of the second admission are reported in Table 3.

**Table 3:** Most significant blood tests at the end of the second admission ALT: Alanine Transaminase; AST: Aspartate Transaminase; Gamma-GT: Gamma-Glutamyl Transpeptidase; Ca 19.9: Carbohydrate antigen 19-9; LDL: Low-Density Lipoprotein

Test	Results	Reference values
Total bilirubin	2.4 mg/dL	< 1.1 mg/dL
Direct bilirubin	1.1 mg/dL	< 0.25 mg/dL
ALT	13 UI/L	< 40 UI/L
AST	25 UI/L	< 40 UI/L
Alkaline phosphatase	171 UI/L	30-130 UI/L
Gamma-GT	93 UI/L	< 38 UI/L
Ca 19.9	36.5 UI/mL	< 38 UI/ml
Blood glucose	105 mg/dL	65-110 mg/dL
Total cholesterol	137 mg/mL	< 200 mg/dL
LDL cholesterol	99 mg/dL	< 100 mg/dL
Triglycerides	190 mg/dL	< 150 mg/dL
Hemoglobin	10.7 g/dL	12-16 g/dL
Creatinine	0.62 mg/dL	0.44-0.95 mg/dL

Five years after surgery, she is still doing well with annual serial checkups showing CMCT scans with no signs of disease recurrence and normal blood tests, including liver function tests and Ca 19.9. Despite a demanding glycemic control with large changes in blood glucose levels, a recent HbA1c level was 54 mmol/mol.

## Discussion

Our case report describes an elderly patient in good health, with pancreatic cysts for at least six years, who presented with NODM at the age of 91. One month later, she had complaints of weight loss and symptoms of obstructive jaundice and an abdomen CMCT evidenced a nodule, suggestive of a malignant neoplastic lesion at the pancreatic head, in a site corresponding to that of a type 1-IPMN, showed six years earlier by CMCT. Clinical and laboratory data strongly support that PDAC manifested about one month after the diagnosis of NODM. The tumor expansion to the head of the pancreas rapidly caused compression on the choledochus resulting in early signs of obstructive jaundice. A timely hospitalization with execution of abdomen CMCT, blood indices of cholestasis, and oncological marker Ca 19.9 moved us towards the high probability diagnosis of pancreatic cancer. Despite the lack of a histological diagnosis, we were led to suggest the possibility of a radical surgery of pancreatic exeresis, in consideration of the absence of metastases, signs of local vascular invasion, and comorbidities in a patient with a high KPSI but with a rapid worsening of the clinical picture. Histological examination confirmed the presence of a PDAC. The success of the surgical intervention was confirmed by the absence of complications both in the post-operative and in the long-term course.

Keeping in mind the presence of pancreatic cysts highlighted by the abdomen US and the diagnosis of NODM in an elderly woman during the first hospitalization, it could be speculated that an in-depth abdomen CMCT performed at that time would have allowed finding a pancreatic nodule suggestive of malignant nature even before the onset of PDAC symptoms.

The clinical course of our case is in agreement with what is reported in two recent reviews [10,13]. They dwell extensively on the goal of early detection of PDAC. Various subgroups at higher than average risk for PDAC have been identified, including those with familial risk due to germline mutations, a history of pancreatitis, patients with mucinous pancreatic cysts, and elderly patients with NODM. In particular, it is stressed that NODM, weight loss, and jaundice indicate a considerably increased risk of developing PDAC. Individuals with NODM are the highest risk group for sporadic PDAC [13] and NODM can be considered an early warning sign of PDAC [7].

The unexpected long duration of survival of our patient is not supported by the results of the following studies. The first one evaluated over 2500 patients with PDAC who underwent total pancreatectomy, with staging and survival data. Median overall survival was 15 months, with one, three, and five-year survival rates of 60%, 22%, and 13%, respectively [14]. Johnston et al. concluded that although total pancreatectomy is a reasonable option for selected patients with PDAC, the survival of the entire group was limited [14]. In the second study, the 2019 Polish guidelines of PDAC emphasize that the long-term results of surgical treatment of PDAC are still not satisfactory. In patients who underwent radical surgery, the median overall survival was 14-17 months, and five-year survival was observed in only 10-27% [15]. The conclusions of the two aforementioned studies lead us to believe that the prognosis of our case is still very comforting.

Conversely, another retrospective study supports our surgical choice. The study aimed to compare the prognostic outcomes of elderly patients (aged ≥ 75 years) with resectable pancreatic cancer. Surgical resection resulted in a better prognosis than non-surgical resection (two-year survival rate, 40.7% vs. 0%) except for patients with a high Charlson comorbidity index. So, an aggressive surgical approach seems to be beneficial for elderly patients with resectable pancreatic cancer [16].

Finally, although for patients with macroscopically radically resected stage IA-III PDAC (R0-R1) and KPSI of at least 50, adjuvant chemotherapy with gemcitabine and capecitabine may be considered [17], in virtue of the age of our patient the benefit-risk ratio ruled out this treatment.

## Conclusions

Our case report highlights the importance to seek the presence of a PDAC in every case of NODM in patients older than 50 years because individuals with NODM are the highest risk group for sporadic PDAC, especially if they carry pancreatic cysts. At the time of the first discharge of our patient, the unusual late onset of NODM prompted us to carry out a close clinical follow-up, which allowed us to promptly search for the causes of the sudden obstructive jaundice that could have heralded a PDAC.

Finally, early diagnosis played as important a role as successful early radical pancreatoduodenectomy in bringing about such a long and extraordinary survival of a PDAC. In this regard, our case represents an exception to the results of large and comprehensive PDAC survival studies.
